# Factors influencing nurses’ satisfaction to online learning approach: a cross ICU analysis

**DOI:** 10.1186/s12912-024-02194-3

**Published:** 2024-08-12

**Authors:** Shu-Yen Lee, Ching-Yi Chang

**Affiliations:** 1 Department of Nursing, Tri-Service General Hospital, Taipei City, Taiwan; 2 School of Nursing, National Defense Medical Center, Taipei City, Taiwan; 3 School of Nursing, College of Nursing, Taipei Medical University, 250 Wuxing Street, Taipei City, 11031 Taiwan

**Keywords:** Mobile learning, Mobile e-learning, Nurses, Online learning, Professional training

## Abstract

**Background:**

Given the increasing complexity of illnesses and rapid pace of technological advancements in professional training, it is vital to offer nurses ample opportunities to hone their clinical expertise and skills, particularly in ensuring the delivery of premier medical care. This study aimed to determine the factors and predictors influencing nurses’ satisfaction with adopting mobile learning approaches in intensive care unit healthcare settings. Additionally, it sought to investigate the applicability of the technology acceptance model in explaining their inclinations and validating the measurement scales employed in the research.

**Methods:**

The study employed a cross-sectional survey research design, utilizing a technology acceptance questionnaire and a learning satisfaction questionnaire. The survey was conducted in six intensive care unit departments. A total of 212 participants completed the survey as the primary instrument. Rigorous assessments were conducted to establish the content validity and ensure instrument reliability.

**Results:**

The findings demonstrated that perceived usefulness was the most influential factor affecting nurses’ intentions to embrace mobile learning approaches, with perceived ease of use emerging as the principal determinant of perceived usefulness.

**Conclusions:**

Incorporating mobile learning methodologies is paramount to increasing the calibration of professional nursing education programs. By effectively integrating digital information technology and tools, nursing educators can overcome teaching challenges, deliver innovative clinical nursing education content through mobile learning approaches, and foster optimal development in the field.

## Introduction

Patient safety considerations arise from high-quality nursing work. Therefore, improving nursing satisfaction is essential. To provide a satisfactory working environment for all aspects of nursing, managers must constantly arrange professional nursing knowledge and skills related to teaching courses. Scholars have also mentioned that improving nursing learning satisfaction can improve work efficiency and quality of care [1]. The most effective way to improve nursing learning satisfaction is to introduce technology into professional nursing knowledge and skills related to teaching courses.

In the context of the coronavirus disease 2019 (COVID-19) pandemic, information technology (IT) is necessary to enable professional staff to continue their education without shift limits and ubiquitous learning with no time limit, as well as to influence their learning satisfaction while improving their professional knowledge and skills [1, 2]. With the rapid development of IT, the cost benefits of utilizing mobile e-learning are particularly noteworthy, especially during the COVID-19 era, as it promotes uninterrupted continuous education, conveys the latest evidence-based medical care information instantaneously, reduces the risk of infection, enhances the quality of care, and offers opportunities for contactless learning [3]. Thus, nurses’ daily clinical work must adapt to medical innovations. Researchers have also highlighted that professional staff with sufficient IT literacy can appropriately use e-commerce platforms, notice, and reduce the incidence of abnormal events, and improve patients’ self-confidence through their care [4, 5]. The goal of introducing IT into hospitals is to improve the quality of medical care, reduce medical care costs, and enhance the effectiveness of clinical medical work [6]. The Technology Informatics Guiding Education Reform initiative recommends that all professional staff cultivate informatics skills and learn how to apply information from evidence and research for practical decision making [7].

## Constructs in the extended TAM

Scholars have pointed out that nurses’ satisfaction with and application of the technology acceptance model (TAM) have demonstrated significant effectiveness in various healthcare settings [8]. Studies have consistently shown that technology acceptance and utilisation are crucial for nurses’ learning satisfaction. By implementing technologies that align with nurses’ needs and preferences, organisations can enhance their job and learning satisfaction and improve their overall work experience [9]. When nurses perceive technology as useful, user-friendly, and supportive of their learning tasks, it increases learning satisfaction while simultaneously contributing to increased retention rates, enhanced productivity, and improved patient outcomes [10]. To examine the perceptions of the clinical environment and explore the factors influencing behavioural intentions, researchers have employed the TAM to study the acceptance of developing blended e-learning systems [11].

The TAM is a tool proposed by Davis to evaluate or predict user acceptance of new technical systems [12]. It is the most widely used technology-based theory in the informatics field. It consists of three main variables that affect an individual’s technology use: perceived convenience, factors that influence the use of these variables, and perceived usefulness (e.g., training, development processes, and technology system characteristics). The two dimensions of perceived usefulness (PU) and perceived ease of use (PEOU) can help explain or predict decision-making and intentions during IT adoption. Venkatesh and Davis determined that the TAM is a mature theoretical model widely used in scientific and technological information systems research [12]. Scholars have further proposed that TAM is an IT framework for understanding user acceptance and willingness to use technology. Researchers have also extended the original TAM to examine the impact of student satisfaction and e-learning on technology acceptance. For example, scholars have extended the TAM to include self-efficacy, perceived satisfaction, and learning methods [13, 14]. The TAM extension was found to be most suitable. Self-efficacy, subjective norms, enjoyment, computer anxiety, and experience are the most commonly used external factors in the TAM. Scholars have also identified these factors in student perceptions of e-learning and proposed a general extension of the TAM [13]. This study aims to include consistent TAM behaviours and meaningful learning in digital technology.

## Perceived usefulness (PU), perceived ease of use (PEOU), and learning satisfaction

Tubaishat described the PU and PEOU of electronic health records among nurses [15]. Williamson and Muckle applied the TAM to learners’ perceptions of the technology used in nursing education [16]. In addition, Baysari et al. explored the impact of user experience with a longitudinal study using TAM to verify the computerized physician order entry system and mistake prevention tools in clinical situations at a paediatric hospital [17]. Bester et al. demonstrated that online learning promotes the ability of nurses to use information communication technology [18]. Furthermore, Nagy used the TAM to effectively evaluate online video usage and the learning satisfaction of nursing staff [19]. Pei-Ying, Chen-Shie, and Pei-Hung implemented TAM via a multilevel interactive nursing quality control setup and investigated learning satisfaction using the audits of nursing quality management [20]. Scholars identified that PU and PEOU learners’ trust sentiments played a significant role in increasing learners’ perceived usefulness and learning satisfaction towards apparel mobile learning [15, 18, 20].

**Fig. 1:**
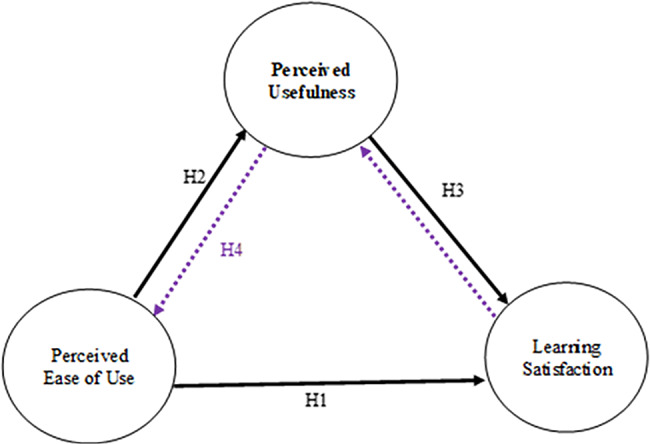
Technology acceptance model for the mobile e-learning system

Based on the above evidence, the study analyses the effectiveness of ICU nurses’ continuing education in e-learning system activities through mobile learning based on nurses’ self-reported perceptions via the extended TAM framework and investigates the acceptance of mobile e-learning systems (Fig. 1). We examined the demographic data and behavioural intent of participating ICU nurses, and the PU and PEOU of the mobile e-learning system to improve their professional knowledge. We propose four research hypotheses: (H1) PEOU can significantly affect learning satisfaction; (H2) PEOU can significantly affect PU; (H3) PU can significantly affect learning satisfaction; and (H4) PEOU can significantly affect learning satisfaction through PU.

## Methods

### Study design

To investigate the time required for ICU nurses to complete relevant continuous education content, the study conducted a cross-sectional survey during two semesters of an academic year (i.e., the 2022–2023 academic year). Cross-sectional surveys are commonly used to gather data from a sample of individuals at specific points in time [21]. In the context of nurses’ perceptions, learning satisfaction, and behavioural intentions, cross-sectional surveys can be a valuable research method for several reasons, such as being well-suited for exploratory and descriptive analyses. In addition, researchers can analyse the collected data to identify patterns, trends, and associations between variables of interest as well as help identify factors and provide insights for future research or interventions [21]. Hence, this survey aimed to conveniently recruit nurses from various departments within medical centres, including internal medicine and cardiac-care centres, surgery and neural care centres, cardiovascular surgery departments, and trauma care centres. This study used course content on the essential core competencies of nurses in care centres (e.g., patient physical assessment, acute and critical drugs, and disease severity assessment). The required core content lists the course topics using mobile learning systems. Mobile learning systems allow learners to access educational resources and participate in interactive activities through mobile devices, thereby enhancing their flexibility and accessibility. Moreover, a mobile learning system can support learning in a clinical environment, and its impact on learners’ perceptions and behavioural intentions can be investigated.

### Participants

The study’s demographic questionnaire was created by the researcher to ascertain sociodemographic profiles to provide important information and help with the inclusion and exclusion of participants. For example, factors, such as work years, degree grades, nurse levels (N1–N4), medical centre category, religion, and the number of children, were included. Nurse levels were based on the work of Hsu and Kernohan [22], which detailed core nurse competency levels (N1–N4) for registered nurses (RNs). The nursing profession in Taiwan has developed a clinical ladder system that categorizes hospital nurses into four levels: N1–N4. A higher level within this system indicates greater professional expertise in the nursing field. The inclusion criterion was nurses aged > 20 years in the ICU. Exclusion criteria were current non-workers and clinical nursing teachers in the ICU. A total of 222 participants from six intensive care unit departments participated in the study. Two left, eight did not complete the questionnaire, and 212 participants completed the study and survey. The 212 participants were volunteers working at the medical centres in northern Taiwan, including the internal medicine and cardiac-care centre, a surgery and neural care centre, a cardiovascular surgery, and a trauma care centre. According to the literature, the minimum ratio of model variables to the number of samples in SEM analysis should be 1:10, and 200 samples is a reasonable number of samples to avoid overexpansion [23]. In addition, a sample size > 500 may lead to the chi-square value being severely inflated, resulting in a poor model fit [24]. Conspicuously large sample sizes have also been associated with slightly negative values of chi-square bias [25]. Our 212 participants thus represented a suitable sample size [26].

### Procedures

A human experimentation ethics committee reviewed, approved, and accepted the protocol after obtaining consent and signed consent forms from the participating ICU nurses. This study was approved by the Institutional Review Board of the Tri-Service General Hospital, National Defense Medical Center (approval number: TSGBIRB:1-110-05-072). Each participant who volunteered to participate in the survey was required to complete the survey within one month after completing the study.

### Materials and instruments

In this study, the technology acceptance questionnaire, which was adapted from Hwang et al. [27], consisted of 13 items and adopted a 5-point Likert scale, with 5 indicating strong agreement and 1 indicating strong disagreement. The original Cronbach’s alpha value was 0.67, and the test-retest Cronbach’s alpha value was 0.75. The scale included 13 items measuring PU (six items) and PEOU (seven items) to explore the cognitive usefulness and cognitive ease of use of remote learning via the mobile e-learning system according to ICU nurses.

The learning satisfaction questionnaire adapted from Chu et al. [28] consisted of 6 items and used a 5-point Likert scale, with 5 indicating strong agreement and 1 indicating strong disagreement. The original Cronbach’s alpha value was 0.72, and the test-retest Cronbach’s alpha value was 0.91, which indicates sufficient reliability for evaluating perceptions about learning activities and attitudes. This scale employs three subscales to examine learning activity: experience, using a technology-based mobile learning system, and satisfaction with the learning method.

### Data analysis

Demographic information and quantitative data analysis were performed using IBM descriptive statistical software SPSS Statistics v. 22 (IBM, Armonk, NY). We used structural equation modelling (SEM) to verify the structure of the intermediary effect model, to understand the potential significance of variables, to estimate and verify the hypothesized relationships, and to collect data for verification. We used IBM AMOS 22.0 software (IBM, Armonk, NY) and analysed correlations between observed variables from the covariance matrix to test model fits. Prior to SEM estimation, hypothetical theoretical models were established. The SEM model included a measurement model and a structural model. The measurement model examined the relationship between the observed variables and the potential model, and the structural model examined any causal relationship between potential variables.

## Results

### Reliability and validity analysis

The analytical quality of the measurement model is based on the construct validity of the test model, which includes convergent and discriminant validity. Discriminant validity is considered to be satisfied if the square root of the average variance is greater than the correlation coefficients of each dimension. Table 1 presents the details of this analysis and shows that all constructs satisfied this criterion. λ of all observed variables and corresponding latent variables ranged from 0.84 to 0.94, exceeding the required threshold of 0.70, and R^2^ was between 0.71 and 0.88, which met the requirement that the reliability of potential individual observation variables should be greater than 0.50 [29]. The construction reliability of latent variables ranged from 0.96 to 0.97, exceeding the required threshold of 0.60; the average variation extraction of latent variables ranged from 0.79 to 0.83, exceeding the required threshold of 0.50, indicating that the contribution of observed latent variables to variance was much larger than the contribution of errors and suggesting that this scale had good convergent validity [30]. The composite reliability (CR) of each scale was larger than 0.7, indicating acceptable reliability of the mode structure [30]. Component reliability, construct validity, and discriminant validities were acceptable as well. The range of average variance extracted (AVE), indicative of the explanatory power of variables for the variance of each measuring item, was greater than 0.5 (between 0.79 and 0.83). Overall, these values suggested that the model was suitably explanatory for the investigated question and performed well.

**Table 1:** Discriminant and convergent validity of the model constructs

	Observed variables	λ	t	*R* ^2^	CR	AVE(%)
PEOU	PEOU1	0.88	---	0.77	0.97	0.83
	PEOU2	0.89	19.34***	0.80		
	PEOU 3	0.91	20.14***	0.82		
	PEOU 4	0.92	20.66***	0.84		
	PEOU 5	0.94	21.96***	0.88		
	PEOU 6	0.91	20.21***	0.83		
	PEOU 7	0.93	21.16***	0.86		
PU	PU 1	0.89	---	0.79	0.96	0.81
	PU2	0.91	20.75***	0.83		
	PU 3	0.86	18.35***	0.75		
	PU 4	0.90	20.18***	0.81		
	PU 5	0.92	21.41***	0.85		
	PU 6	0.92	21.34***	0.84		
Learning Satisfaction (LS)	LS1	0.88	---	0.77	0.97	0.79
	LS2	0.85	21.01***	0.71		
	LS3	0.87	18.28***	0.76		
	LS4	0.84	17.04***	0.71		
	LS5	0.90	19.58***	0.82		
	LS6	0.94	21.43***	0.88		
	LS7	0.91	19.74***	0.82		
	LS8	0.90	19.64***	0.81		
	LS9	0.93	20.99***	0.86		

CR: Composite reliability; AVE: Average variance extracted; *p < .05, **p < .01, ***p < .001

### Results of the model fit and research hypotheses

Based on previous studies, we also confirmed the reliability of the scales used to measure these variables through a test-retest analysis [31, 32]. To test the model validity further, we carried out a confirmatory factor analysis based on Bollen-Stine bootstrapping [33]. The model satisfied various model fit parameters (Table 2): Bollen-Stine-corrected chi-square, 469.003 (df = 200); χ^2^ per degree of freedom, 2.345; GFI, 0.84; AGFI, 0.80; CFI, 0.96; NNFI (TLI), 0.95; and RMSEA, 0.08. These results indicated that the model fit the study’s data well [33–35] and suggested suitable reliability in accessing the participants’ perceptions of PEOU, PU, and learning satisfaction.

**Table 2:** Model fit parameters

Criterion		Standard	
Df	--	200	--
GFI	≥ 0.90	0.841	acceptable
AGFI	≥ 0.90	0.799	acceptable
SRMR	≤ 0.08	0.025	good
RMSEA	≤ 0.08	0.080	good
NFI	≥ 0.90	0.932	good
NNFI	≥ 0.90	0.953	good
RFI	≥ 0.90	0.921	good
IFI	≥ 0.90	0.960	good
CFI	≥ 0.90	0.959	good
PGFI	≥ 0.50	0.665	good
PNFI	≥ 0.50	0.807	good
PCFI	≥ 0.50	0.831	good
Likelihood ratio χ^2^/*df*	≤ 3	2.345	good

GFI: Goodness of fit; AGFI: Adjusted goodness of fit; SRMR: Standardized root mean square; RMSEA: Root mean square error of approximation; NFI: Normed fit index; NNFI: Non normed fit index; RFI: Relative fit index; IFI: Incremental fit index; CFI: Comparative fit index; PGFI: Parsimonious goodness of fit ; PNFI: Parsimony-adjusted measures index; PCFI: Parsimonious comparative fit index; *p < .05, **p < .01, ***p < .001

### Response hypothesis 1: PEOU can significantly affect learning satisfaction

Based on Baron and Kenny [36], the intermediary effect test procedure employed the bootstrapping method proposed by Shrout and Bolger to calculate indirect sampling effects by constructing a new confidence interval during re-sampling [37]. If the 95% confidence interval does not include 0, the indirect effect is significant. We applied the three steps described by Baron and Kenny to confirm the existence of moderating effects [35].

Table 3 shows that the PEOU of the mobile learning system showed a significant positive effect on learning (total effect; *β* = 0.79, *p* < .001) with a positive regression coefficient, indicating that PEOU can significantly and positively affect learning satisfaction. This outcome also shows that with higher PEOU of the mobile e-learning system, learning satisfaction is higher. Therefore, our results support Hypothesis 1.

**Table 3:** Direct effect coefficient analysis of the moderation effect structural equation model (*N* = 212)

Hypothesis	Path relationship	Unstandardized coefficient	Standard error	t	*p*	Standardized coefficient	Result
H1: Hypothesis 1	PEOU → satisfaction (Total effect)	0.77	0.06	12.87***	< 0.001	**0.79**	Positive
H2: Hypothesis 2	PEOU → PU	0.89	0.06	15.34***	< 0.001	**0.88**	Positive
H3: Hypothesis 3	PU → satisfaction	0.70	0.09	7.62***	< 0.001	**0.73**	Positive
H4: Hypothesis 4	Indirect effect	0.62	0.15	0.37–0.93*	0.001	**0.64**	Positive
	Direct effect	0.15	0.09	1.71	0.088	**0.15**	Negative

PEOU, perceived ease of use; PU, perceived usefulness; *p < .05, **p < .01, ***p < .001

### Response hypothesis 2: PEOU can significantly affect PU

For Hypothesis 2, the results show that PEOU of the mobile e-learning system has a significant positive effect on PU (*β* = 0.88, *p* < .001), and the regression coefficient was positive, implying that PEOU can significantly and positively affect PU. This outcome means that with higher PEOU of the mobile e-learning system, PU is also higher. Therefore, our results support Hypothesis 2.

### Response hypothesis 3: PU can significantly affect learning satisfaction

The results concerning Hypothesis 3 show that the PU of the mobile e-learning system has a significantly positive effect on learning satisfaction (*β* = 0.73, *p* < .001) with a positive regression coefficient, indicating that PU can significantly and positively affect learning satisfaction. This also means that with a higher PU, the learning satisfaction from using the mobile e-learning system is also higher. Therefore, our results support Hypothesis 3.

### Response hypothesis 4: PEOU can significantly affect learning satisfaction through PU

To examine Hypothesis 4, bootstrapping with 1,000 repeated samples was used to verify the significance of the indirect effect of PU on PEOU and learning satisfaction. The results showed that the 95% confidence interval of the bias-corrected percentile from the bootstrap analysis was between 0.37 and 0.93, which does not include 0. Therefore, the indirect effect (0.64) was significant and the moderating effect was verified. The effect of PEOU on learning satisfaction was reduced from 0.79 to 0.15, which was not significant. This result indicates complete mediation. In summary, the greater the PEOU, the less likely learning satisfaction will directly affect the results. In contrast, with increasing PEOU, increasing PU can further affect learning satisfaction.

## Discussion

The findings provide insights into the analytical quality of the measurement model, its construct validity, and the relationship between the variables of interest. The measurement model demonstrates satisfactory discriminant and convergent validity. All constructs met the criterion for discriminant validity, as indicated by the square root of the average variance, which exceeded the correlation coefficients for each dimension.

The observed and corresponding latent variables have high factor loadings (λ), indicating strong relationships. The reliability of the individual observation variables (R2) exceeds the required threshold of 0.50, indicating good reliability. The latent variables demonstrated high construction reliability and average variance extraction, exceeding the thresholds of 0.60 and 0.50, respectively. This suggests that the observed latent variables contributed significantly to the variance, thereby validating the scale’s convergent validity. The composite reliability (CR) of each scale was acceptable. Furthermore, confirmatory factor analysis supported the validity of the model, with the model fitting the data well, based on various fit parameters. This suggests suitable reliability in assessing the participants’ perceived ease of use (PEOU), perceived usefulness (PU), and learning satisfaction.

Regarding the study’s SEM analysis results, Hypothesis 1 indicates that PEOU significantly affects learning satisfaction. A higher PEOU for mobile learning systems leads to increased learning satisfaction. Hypothesis 2 was also supported, as PEOU significantly and positively affected PU. A higher PEOU for a mobile learning system corresponds to a higher PU. Hypothesis 3 is also supported, indicating that PU significantly affects learning satisfaction. A higher PU from using a mobile learning system leads to increased learning satisfaction. Using the TAM framework, we found that ICU nurses perceived remote learning technology to be useful and easy to use, highlighting their enthusiasm for mobile e-learning systems.

Hypothesis 4 suggested that PEOU indirectly affects learning satisfaction. Bootstrapping analysis confirmed the significant indirect effect of PU on PEOU and learning satisfaction. This implies that PU partially mediates the relationship between PEOU and learning satisfaction. As PEOU increases, PU further enhances learning satisfaction, suggesting a complete mediating effect.

Satisfaction is an indicator of intention to use. According to the TAM, high PU and PEOU influence satisfaction and behavioural intention to use, which in turn affects concrete practice. Our analysis showed that ICU nurses were satisfied with the mobile e-learning system and identified it as beneficial and comprehensible. This result echoes Williamson and Muckle [16], who pointed out that staff uses technology more when they perceive it to be useful and easy to use. Similarly, learners voluntarily agree to use technology according to the extent of their information literacy.

TAM outlines PEOU and PU, which are the core variables of our model [15]. Nagy claimed that PU is directly affected by PEOU [19]. As our study shows, SEM indicated that PEOU did have a major direct effect on PU. Meanwhile, PU can mediate learning satisfaction. Our findings also indicate that PEOU had a significant direct effect on learning satisfaction. The mobile e-learning system was perceived as comprehensible, encouraged attentiveness to any schedule changes via the system, and provided an opportunity to record information and upload materials. Our results suggest that the PEOU of mobile e-learning system use improves the ease of learning and can enhance ubiquitous learning, particularly beneficial during the current pandemic. This style of learning may also inspire learners with inadequate internet connection. According to Salloum et al. [38], learners tend to find material online, use mobile e-learning platforms, and communicate with others to aid them in their learning. One challenge of using different digital platforms to learn is internet connection issues. Therefore, interactive, accessible methods and demonstrations that rely on the internet may be a challenge for learners with weaker internet connections. In future research, different digital platforms could be evaluated via TAM. For instance, studies in different professional training areas might be conducted on virtual reality, augmented reality, game-based mobile learning, and investigation of learners’ learning self-efficacy and learning attitude. Such applications would provide further contributions and insights for the professional training field.

Overall, the findings align with the prior literature and provide evidence for the relationships between PEOU, PU, and learning satisfaction in mobile e-learning systems. These results contribute to our understanding of the factors influencing learner satisfaction and highlight the importance of PEOU and PU in shaping learning outcomes. All four hypotheses were supported by our results, which described the effects of remote learning on the learning satisfaction of ICU nurses using the TAM. Our results support the notion that mobile e-learning systems can enhance progress in the medical industry. The TAM for model learning has not yet been tested in ICU nurses’ education during the COVID-19 pandemic [39, 40]. According to Choi et al. [41], nurses must be trained in problem-solving skills and this should include information literacy training in future nursing courses, especially with increasing technological advancements in health care. This also affects the responsibilities of medical education managers who should employ innovative teaching and learning strategies using mobile e-learning systems to shape clinical digital learning environments and healthcare education settings.

In summary, SEM showed that PU and PEOU had a significant direct impact on learning satisfaction. Currently, distance learning methods using mobile e-learning systems are being used to access different professional classes online for continuing education. Remote learning is very useful during COVID-19 pandemic because it limits potential exposure to infection. These systems could also be useful to learners across different disciplines and countries. Therefore, mobile e-learning systems are a useful and safe alternative to traditional learning.

### Limitations

While this study addresses several apparent research gaps and successfully extends TAM to medical contexts, it is subject to some potential limitations that need to be identified and discussed. First, our study specifically examined the e-learning outcomes of ICU nurses, and the theoretical implications of the TAM should be explored in future research. For instance, future studies could investigate the application of the TAM in conjunction with various technologies such as augmented reality (AR), virtual reality (VR), and game-based learning, targeting diverse nursing or medical staff for professional training purposes. Next, we did not investigate whether ICU nurses’ work years affected our sample-related internal factors for TAM that may include different nurse levels (N1–N4). Future investigations might consider carrying out similar analyses at each of these individual levels. Third, our study considered the impact of moderating factors in an e-learning environment in a single setup. Future research should extend this question to a variety of different work situations, such as conducting TAM in different professional training areas, at different nurse levels (N1–N4), and focusing on virtual reality, augmented reality, or game-based mobile learning.

## Conclusion

Based on the findings of the study, nurses’ continuing education activities via mobile e-learning systems enhance not only technology acceptance but also learning satisfaction. Thus, medical educators should be promoting learning effectiveness and using available educational methods and technology to attract learners so that they can study safely and learn to provide high-quality, professional, and safe care. The results of this study can help direct the integration of the mobile e-learning systems into innovative teaching curricula in the medical field. Teachers and learners should be supported in effectively using the mobile e-learning systems to transmit professional knowledge and models.

During the COVID-19 pandemic, developing a continuous education system has been challenging. People around the world are learning how to transform traditional learning methods to fully online digital learning systems. In addition, many paper materials and textbooks take too long to be updated, while with the real-time spread of mobile learning and digital textbooks, online learning can provide opportunities for newer and more sustainable learning models. As IT continues to develop, both teachers and learners need to adapt to the expansion of IT, which can be combined with medical professional education and training programmes, even after the COVID-19 pandemic. Therefore, the application of the TAM framework combined with professional curriculum design can help cultivate core IT literacy, enhance the critical thinking and problem-solving skills of clinical medical care workers, and can meet continuing education goals for future educators.

## Data Availability

The datasets used and/or analysed during the current study available from the corresponding author on reasonable request.

## References

[1] Drexler D. Using a nursing professional governance approach to improve nurse satisfaction and participation with health information technology. Nurse Lead. 2020;18(3):276–80. 10.1016/j.mnl.2020.03.003

[2] Greener S. Student wellbeing in the learning zone. Interact Learn Environ. 2020;28(7):806–7. 10.1080/10494820.2020.1832718

[3] Kondylakis H, Katehakis DG, Kouroubali A, Logothetidis F, Triantafyllidis A, Kalamaras I, Tzovaras D. COVID-19 mobile apps: a systematic review of the literature. J Med Int Res. 2020;22(12):e23170. 10.2196/23170 PMC7732358 33197234

[4] Goh J, Truman B, Barber D. Exploring individual differences as factors to maximize interactive learning environments for future learning. Interact Learn Environ. 2019;27(4):497–507. 10.1080/10494820.2018.1484775

[5] Simamora RH. Socialization of information technology utilization and knowledge of information system effectiveness at hospital nurses in Medan, north Sumatra. Editorial Preface Desk Managing Editor 10(9) (2019).

[6] Ng YC, Alexander S, Frith KH. Integration of mobile health applications in health information technology initiatives: expanding opportunities for nurse participation in population health. CIN-Comput Inf Nurs. 2018;36(5):209–13. 10.1097/CIN.0000000000000445 29734236

[7] McBride S, Hoelscher SH, Bumpus S, Mitchell MB, Tietze M. Crisis documentation strategies to reduce burden of documentation during the pandemic: Texas’ pilot to generate consensus, CIN-Comput. Inf Nurs. 2021;39(10):524–6. 10.1097/CIN.0000000000000842 PMC8594409 34623336

[8] Alanazi B, Butler-Henderson K, Alanazi M. Perceptions of healthcare professionals about the adoption and use of EHR in gulf cooperation council countries: a systematic review. BMJ Health Care Inf. 2020;27(1):e100099. 10.1136/bmjhci-2019-100099. 10.1136/bmjhci-2019-100099 PMC7062356 31924667

[9] Hebb A, Kistler M, George E, Zamboni B. Satisfaction and technology acceptance of staff regarding use of continuous video monitoring in comparison with sitters. J Nurs Adm. 2021;51(2):60–2. 33449593 10.1097/NNA.0000000000000970

[10] Marcus JA, Clark PC. Psychometrics of nurses’ perceptions of technology effectiveness scale. J Contin Educ Nurs. 2021;52(5):248–52. 34038682 10.3928/00220124-20210414-09

[11] Tsai YY, Chao CM, Lin HM, Cheng BW. Nursing staff intentions to continuously use a blended e-learning system from an integrative perspective. Qual Quant. 2018;52(6):2495–513. 10.1007/s11135-017-0540-5

[12] Davis FD. Perceived usefulness, perceived ease of use, and user acceptance of information technology. MIS Q. (1989)319–40.

[13] Lai CY, Lee TY, Lin SC, Lin IH. Applying the technology acceptance model to explore nursing students’ behavioral intention to use nursing information smartphones in a clinical setting, CIN-Comput. Inf Nurs. 2022;40(7):506–12. 10.1097/CIN.0000000000000853 35120371

[14] Kalayou MH, Endehabtu BF, Tilahun B. The applicability of the modified technology acceptance model (TAM) on the sustainable adoption of eHealth systems in resource-limited settings. J Multidiscip Healthc (2020)1827–37. 10.2147/JMDH.S284973 PMC7721313 33299320

[15] Tubaishat A. Perceived usefulness and perceived ease of use of electronic health records among nurses: application of technology acceptance model. Inf Health Soc Care. 2018;43(4):379–89. 10.1080/17538157.2017.1363761 28920708

[16] Williamson KM, Muckle J. Students’ perception of technology use in nursing education. CIN-Comput Inf Nurs. 2018;36(2):70–6. 10.1097/CIN.0000000000000396 29084028

[17] Baysari MT, Hardie RA, Lake R, Richardson L, McCullagh C, Gardo A, Westbrook J. Longitudinal study of user experiences of a CPOE system in a pediatric hospital. Int J Med Inf. 2018;109:5–14. 10.1016/j.ijmedinf.2017.10.018 29195706

[18] Bester P, Smit K, De Beer M, Myburgh PH. When online learning becomes compulsory: student nurses’ adoption of information communication technology in a private nursing education institution. Curationis. 2021;44(1):2152. 10.4102/curationis.v44i1.2152. 34797105 10.4102/curationis.v44i1.2152 PMC8603156

[19] Nagy JT. Evaluation of online video usage and learning satisfaction: an extension of the technology acceptance model. Int Rev Res Open Distrib Learn. 2018;19(1). 10.19173/irrodl.v19i1. 2886CopiedAn error has occurred.

[20] Pei-Ying KO, Chen-Shie HO, Pei-Hung LIAO. The impact of a multilevel interactive nursing quality control and audit application on nursing quality management. BMC Nurs. 2021;20(1):1–11. 34872533 10.1186/s12912-021-00767-0 PMC8647066

[21] Yu P, Li H, Gagnon MP. Health IT acceptance factors in long-term care facilities: a cross-sectional survey. Int J Med Inf. 2009;78(4):219–29. 10.1016/j.ijmedinf.2008.07.006 18768345

[22] Hsu MY, Kernohan G. Dimensions of hospital nurses’ quality of working life. J Adv Nurs. 2006;54(1):120–31. 16553697 10.1111/j.1365-2648.2006.03788.x

[23] Jackson DL. Revisiting sample size and number of parameter estimates: some support for the N: Q hypothesis. Struct Equ Model. 2003;10(1):128–41. 10.1207/S15328007SEM1001_6

[24] Lei PW, Wu Q. Introduction to structural equation modeling: issues and practical considerations. Educ Meas -Issues Pract. 2007;26(3):33–43. 10.1111/j.1745-3992.2007.00099.x

[25] Smid SC, Winter SD. Dangers of the defaults: a tutorial on the impact of default priors when using bayesian SEM with small samples. Front Psychol. 2020;11:3536. 10.3389/fpsyg.2020.611963 PMC7759471 33362673

[26] Westland JC. Lower bounds on sample size in structural equation modeling. Electron Commer Res Appl. 2010;9(6):476–87. 10.1016/j.elerap.2010.07.003

[27] Hwang GJ, Yang LH, Wang SY. A concept map-embedded educational computer game for improving students’ learning performance in natural science courses. Comput Educ. 2013;69:121–30. 10.1016/j.compedu.2013.07.008

[28] Chu HC, Hwang GJ, Tsai CC, Tseng JC. A two-tier test approach to developing location-aware mobile learning systems for natural science courses. Comput Educ. 2010;55(4):1618–27. 10.1016/j.compedu.2010.07.004

[29] Hair JF, Black WC, Babin BJ, Anderson RE. Multivariate data analysis. Upper Saddle River, NJ: Prentice Hall; 2009.

[30] Fornell C, Larcker DF. Evaluating structural equation models with unobservable variables and measurement error. J Mark Res. 1981;18(1):39–50. 10.1177/002224378101800104

[31] Yu Z. Extending the learning technology acceptance model of WeChat by adding new psychological constructs. J Educ Comput Res. 2020;58(6):1121–43. 10.1177/0735633120923772

[32] Alsalhi NR, Eltahir ME, Al-Qatawneh SS. The effect of blended learning on the achievement of ninth grade students in science and their attitudes towards its use. Heliyon. 2019;5(9):e02424. 10.1016/j.heliyon.2019.e02424. 31535048 10.1016/j.heliyon.2019.e02424 PMC6744605

[33] Icoz K, Sanalan VA, Ozdemir EB, Kaya S, Cakar MA. Using students’ performance to improve ontologies for intelligent e-learning system. Educ Sci -Theory Pract. 2015;15(4):1039–49.

[34] Jackson DL Jr, Gillaspy JA. Purc-Stephenson, reporting practices in confirmatory factor analysis: an overview and some recommendations, Psychol. Methods. 2009;14(1):6. 10.1037/a0014694 19271845

[35] Shah R, Pillai P. Consumer’s environmental concern & its influence on their purchase intention: SEM Approach. Int J Manag. 2012;6(2):24–31.

[36] Baron RM, Kenny DA. The moderator–mediator variable distinction in social psychological research: conceptual, strategic, and statistical considerations. J Pers Soc Psychol. 1986;51(6):1173. 3806354 10.1037/0022-3514.51.6.1173

[37] Shrout PE, Bolger N. Mediation in experimental and nonexperimental studies: new procedures and recommendations. Psychol Methods. 2002;7(4):422. 12530702 10.1037/1082-989X.7.4.422

[38] Salloum SA, Alhamad AQM, Al-Emran M, Monem AA, Shaalan K. Exploring students’ acceptance of e-learning through the development of a comprehensive technology acceptance model. IEEE Access. 2019;7:128445–62. 10.1109/ACCESS.2019.2939467

[39] Hsu HH, Wu YH. Investigation of the effects of a nursing information system by using the technology acceptance model. CIN-Comput Inf Nurs. 2017;35(6):315–22. 10.1097/CIN.0000000000000313 27832033

[40] Fauzi A, Wandira R, Sepri D, Hafid A. Exploring students’ acceptance of Google classroom during the COVID-19 pandemic by using the technology acceptance model in West Sumatera universities. Electron J E-Learn. 2021;19(4):233–40. 10.34190/ejel.19.4.2348

[41] Choi EJ, Park JH, Kang SW. Nursing students’ acceptance intention of a smart device, information literacy, and problem-solving confidence. Healthc. 2021;9(9):1157. 10.3390/healthcare9091157. 10.3390/healthcare9091157 PMC8471261 34574931

